# Digitalization of Indonesian SOEs and Employee Mental Health: Mitigating Digital Anxiety in Finance Functions

**DOI:** 10.12688/f1000research.176773.1

**Published:** 2026-02-17

**Authors:** Kurniasari Novi Hardanti, Sutrisno T, Erwin Saraswati, Arum Prastiwi

**Affiliations:** 1 Department of Accounting, Faculty of Economics and Business, Universitas Brawijaya, Malang, East Java, 65145, Indonesia

**Keywords:** Digital anxiety, organizational climate, regulatory support, and ICT skill

## Abstract

This study investigates factors that mitigate digital anxiety, focusing on organizational climate (digital training, role clarity, teamwork, transformational leadership), regulatory support, and information and communication technology (ICT) skills. Grounded in the Technology-Organization-Environment framework, this research provides a novel contribution by exploring these relationships in the context of state-owned enterprises (SOEs) in Indonesia. Data were collected through a survey targeting finance department employees in SOEs who utilize digital technology in their daily tasks. A total of 270 valid responses were analyzed using SmartPLS. The findings reveal that organizational climate variables along with regulatory support, significantly reduce digital anxiety. Moreover, ICT skills enhance the negative impact of digital training and teamwork on digital anxiety. However, the moderating role of ICT skills in strengthening the effects of role clarity and transformational leadership on digital anxiety was not supported. The practical implications of this study are significant for SOE management. To foster a supportive organizational climate, managers should ensure clearly defined roles through detailed job descriptions and encourage collaborative teamwork. Such measures can help employees address challenges collectively, thereby alleviating digital anxiety. These insights contribute to both academic literature on workplace digital transformation and managerial strategies for employee well-being.

## 1. Introduction

The Ministry of Communication and Informatics of Indonesia released the 2022 digital society index at 37.8 on a scale of 1-100, indicating significant challenges in digital competence among the population. A low IMD score is associated with heightened anxiety regarding the use of digital technologies, which can disrupt daily activities and overall well-being. From a self-regulation perspective, anxiety manifests as feelings of restlessness, worry, or fear ( Wood & Bandura, 1989).

Technological advancements contribute to this anxiety, as they are perceived to mimic human cognitive processes and threaten job security; projections suggest that between 400 to 800 million workers may be displaced by technology by 2030 ( Li & Huang, 2020). While digitalization offers numerous benefits, it also introduces risks that can trigger negative emotional responses such as anxiety ( Pfaffinger et al., 2021). Individuals may respond to such anxiety by either withdrawing or increasing their engagement with technology, a reaction influenced by their adaptive capabilities ( Carver & Scheier, 2021). Despite the pressing nature of digital anxiety as a social issue resulting from digital transformation, it remains underexplored in academic literature.

Digital anxiety represents a significant emotional challenge within corporate environments, providing insights into individual behaviors ( Ashkanasy & Daus, 2002; Bharat, 2011; Repenning et al., 2022). In the financial sector, this form of anxiety arises from the pressures of digital transformation involving AI, IoT, big data analytics, and ICT applications—tools that can enhance financial management but also exacerbate employee fears regarding job security ( Firk et al., 2023; Nugroho, 2019). The presence of digital anxiety can diminish both individual and organizational performance ( Bala & Venkatesh, 2016; Kent et al., 2023; Pfaffinger et al., 2021), leading employees to perceive digitalization as a threat rather than an opportunity for enhancing work quality ( Firk et al., 2023). This emotional state can result in frustration, burnout, loss of motivation, and ultimately disengagement from work processes. Ultimately digital anxiety can be a barrier to digital transformation in finance, but it is still unclear about the level of anxiety in the finance function and how to reduce digital anxiety in employees.

To address these challenges, it is crucial to enhance individual self-efficacy in navigating technology through three key mechanisms: competence development, fostering belief in one’s ability to manage situations, and increasing motivation for specific behaviors ( Wood & Bandura, 1989). This study aims to investigate how these mechanisms interact with four critical organizational climate factors: digital training effectiveness, role clarity, teamwork dynamics, and transformational leadership. Organizational climate is believed to have a direct impact on employee motivation in using a technology ( Madhukar et al., 2017). The implementation of digital transformation is highly dependent on the organizational climate ( Rücker et al., 2021).

This study investigates environmental factors that potentially alleviate digital anxiety, framed within the Technology-Organization-Environment (TOE) model. The TOE framework, introduced by Tornatzky and Fleischer (1990), emphasizes that technological, organizational, and environmental elements significantly impact technology adoption by both organizations and individuals. Environmental factors are crucial for comprehending technology usage ( AlBar & Hoque, 2019). Furthermore, the Theory of Planned Behavior (TPB) effectively elucidates how external environmental aspects influence technology utilization ( Tan & Teo, 2000). The proliferation of technology is facilitated when the requisite technological infrastructure is accessible and user-friendly. Government intervention and leadership are pivotal in promoting innovation diffusion among individuals ( Hai & Kazmi, 2015; Tan & Teo, 1998).

Within the framework of the Technology Acceptance Model (TAM), technological factors play a critical role in user acceptance of information technology ( Davis et al., 1989). TAM effectively addresses how technological aspects can diminish digital anxiety ( Akrong et al., 2022; Gefen & Straub, 2004). The perceived ease of use within TAM correlates closely with users’ ICT (Information and Communication Technology) skills; users expect technology to be intuitive, reflecting their foundational skills.

ICT has revolutionized various life aspects ( Bosamia, 2013; Jandsalar, 2022). However, a significant barrier to successful digital transformation is the scarcity of human resources skilled in ICT. The influence of ICT proficiency on digital anxiety is paramount during digitalization efforts. Extended computer usage enhances user behavior concerning ERP systems in Ghana indicating that increased experience with technology fosters improved engagement ( Akrong et al., 2022). Camilleri (2020) found the same thing, that skills in using a technology will increase user involvement in using e-government systems.

This study addresses a notable gap in research concerning digital anxiety ( Firk et al., 2023) *.* Digital anxiety poses challenges to financial transformation; however, the specific levels of anxiety within finance functions and strategies for alleviating employee concerns remain underexplored. The ongoing digital transformation within SOEs has garnered attention as these entities increasingly leverage digital technologies for operational efficiency and expedited decision-making. State-owned enterprises in Indonesia has adopted blockchain technology across various facets of digital finance—including trade finance, carbon transactions, and public financial management—facilitating secure data exchanges that are anticipated to boost transaction volumes and corporate revenues.

The objectives of this study are threefold: to examine the impact of organizational climate—specifically digital training, role clarity, teamwork, and transformational leadership—on digital anxiety; to assess the influence of regulatory support on digital anxiety; and to evaluate the moderating effect of ICT skills on the relationship between organizational climate factors and digital anxiety.

This study aims to refine existing theories by integrating social cognitive theory, Technology-Organization-Environment, TAM, and TPB into a single model. Practically, the findings serve as valuable insights for systems analysts and organizational management. For IT analysts tasked with implementing information systems within finance departments, it is essential to consider how information technology can enhance job efficiency, accelerate processes, boost productivity, and elevate employee performance. For organizational leaders, this study offers guidance on fostering a positive organizational climate conducive to effective digital transformation initiatives.

## 2. Literature review

### 2.1 Research model and hypothesis

**Figure 1.**
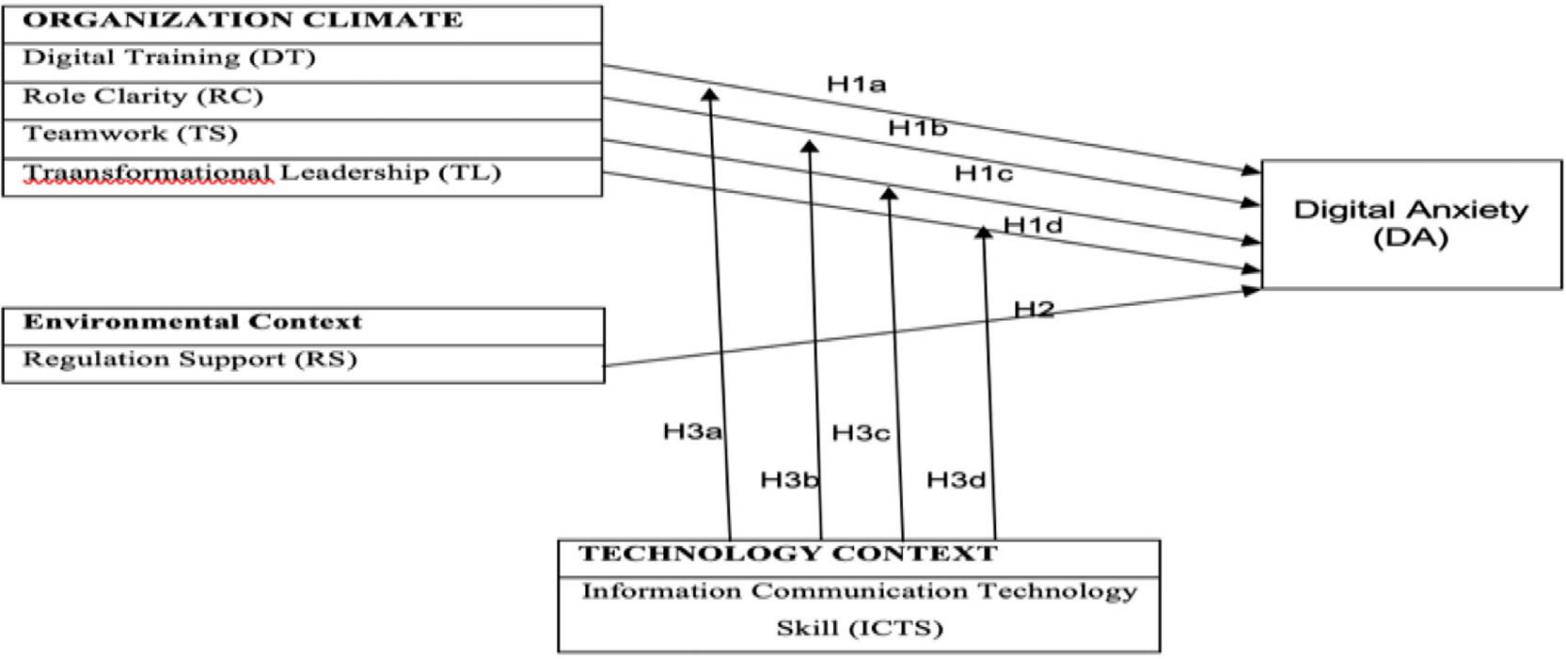
Proposed theoretical framework.

Figure 1 shows the research model developed from the previously described literature. The model in this study seeks to understand the factors that can mitigate digital anxiety. This study develops a social cognitive theory in organizations built by ( Wood & Bandura, 1989). This theory posits that an individual’s self-efficacy is influenced by the surrounding organizational factors. In addition to organizational influences, the Technology-Organization-Environment (TOE) model highlights the importance of environmental and technological factors ( Tornatzky & Fleischer, 1990).

*2.1.1 Digital training hypothesis on digital anxiety*

Competency development is essential for organizational change, aligning with social cognitive theory ( Wood & Bandura, 1989). This theory posits that enhancing competencies within companies can improve understanding of technology usage determinants. Goretzki et al., (2013) emphasize that competency development is crucial for organizational transformation, particularly through digital training. Regular digital training is expected to reduce employees’ digital anxiety.

Training can help employees adapt to change and be positive about the digital transformation that is happening in the finance department. Based on these insights, the following hypothesis is proposed:

H1a: Digital training negatively affects digital anxiety.

*2.1.2 Role clarity hypothesis on digital anxiety*

The second mechanism in social cognitive theory emphasizes the importance of individuals’ belief in their ability to cope with situations ( Wood & Bandura, 1989). This confidence is crucial for self-regulation and emotional responses ( Firk et al., 2023). Beliefs are shaped by the organizational context, as outlined in social cognitive theory ( Wood & Bandura, 1989). Clear role definitions during digital transformation enhance individual confidence. Role clarity is essential for successful technology implementation, as it involves understanding organizational expectations regarding job outcomes (Y. Li et al., 2020). Research indicates that role clarity is linked to higher job satisfaction (J. Li & Huang, 2020; Thangavelu & Sudhahar, 2017). Employees with well-defined goals and responsibilities are more likely to know whom to approach for support and guidance in using new systems. This clarity significantly impacts individual performance. Based on this rationale, the following hypothesis is proposed:

H1b: Role clarity negatively affects digital anxiety.

*2.1.3 Teamwork hypothesis on digital anxiety*

The second mechanism in Social Cognitive Theory relates to the belief that individuals must be able to cope with situations ( Wood & Bandura, 1989). Confidence in managing specific situations is critical for self-regulation and emotional responses ( Firk et al., 2023) and is influenced by the surrounding organizational context ( Wood & Bandura, 1989).

Teamwork plays a crucial role in supporting individuals during digital transformation. It refers to the ability of technology users to receive timely assistance from colleagues, service providers, and the information systems department ( Akrong et al., 2022). Teamwork is fostered through collaboration, mutual support, and shared accountability ( Caesens et al., 2016; Hanaysha & Tahir, 2016). Research demonstrates that teamwork significantly enhances the adoption of information systems ( Costa et al., 2020) and underscores its importance in ERP system implementation ( Almajali et al., 2016). Based on this rationale, the following hypothesis is proposed:

H1c: Teamwork negatively affects digital anxiety.

*2.1.4 Transformational leadership hypothesis on digital anxiety*

The third mechanism in Social Cognitive Theory pertains to employee motivation to achieve specific goals ( Wood & Bandura, 1989). Motivated employees are more likely to engage in required behaviors, thus reducing susceptibility to negative emotions ( Bandura, 1988). Goretzki et al., (2013) highlight that leadership actions—such as storytelling, effective communication, and leading by example—can foster motivation and facilitate organizational change. Transformational leaders can effectively motivate employees to embrace the digital transformation vision, especially in the financially strategic sector, ultimately alleviating digital anxiety. Transformational leadership is inherently about change ( Money, 2017). Transformational leaders can encourage their followers to change their expectations, perceptions, and motivations to work toward common goals. Based on this analysis, the following hypothesis is proposed:

H1d: Transformational leadership negatively affects digital anxiety.

*2.1.5 Hypothesis of regulatory support for digital anxiety*

Regulatory support refers to the policies and regulations that facilitate technology adoption, enhancing the competitiveness of business processes ( Pudjianto et al., 2011). Numerous studies have identified government regulatory support as a key driver of technology adoption ( Amini et al., 2014; Charag et al., 2020; W. C. Chen et al., 2019; Hai & Kazmi, 2015). Baker (2011) indicates that environmental factors positively influence usage behavior, suggesting that greater government support correlates with higher technology adoption rates. The influence of government support formulated and proven by ( Rogers, 1983) in TOE, has been supported by many studies ( AlBar & Hoque, 2019; Junior et al., 2019). Junior et al., (2019) conducted a study of 375 farmers in Brazil who use ERP in their work processes. Based on this analysis, the following hypothesis is proposed:

H2: Regulatory support negatively affects digital anxiety.

*2.1.6 ICT Skills as a moderating variable*

This study proposes that ICT (Information and Communication Technology) skills will moderate the influence between independent variable and dependent variable. Derived from the Technology Acceptance Model ( Davis et al. 1989), ICT skills are crucial for reducing digital anxiety by enhancing perceived ease of use. Proficient ICT skills enable users to navigate technology more comfortably, which is essential for successful digital transformation ( Marston et al., 2011). Research indicates that inadequate ICT skills can lead to decreased motivation and increased anxiety among users ( Lutovac & Manojlov, 2012). Akrong et al., (2022) found that prolonged computer use positively influences system usage behavior. As individuals become more adept at using technology, their self-confidence grows, thereby alleviating anxiety ( Alkhawaja et al., 2021; Iraola-Real et al., 2023). Thus, the following hypothesis is proposed:

H3a: ICT skills strengthen the negative effect of digital training on digital anxiety.

H3b: ICT skills strengthen the negative influence of role clarity on digital anxiety.

H3c: ICT skills strengthen the negative effect of teamwork on digital anxiety.

H3d: ICT skills strengthen the negative influence of transformational leadership on digital anxiety.

## 3. Research methodology

The population used in this study is all individuals working at State-Owned Enterprises in Indonesia, selected for their adaptability to technological changes. State-Owned Enterprises have actively integrated digital technology to enhance operational efficiency and facilitate quicker decision-making. The unit of analysis includes employees from finance, accounting, internal audit, and taxation departments within SOEs undergoing digital transformation. This focus is justified as SOEs are required not only to master digital platforms but also to engage in creative and innovative practices to optimize their functions. A purposive sampling method was employed, selecting non-probability samples based on specific criteria.

Research data were collected through a survey using a questionnaire distributed via Google Forms to gather responses from the finance department regarding digital transformation. The anonymous nature of the survey encouraged open and confidential responses, which is crucial for addressing digital anxiety. Qualitative insights were also incorporated from respondent comments. Prior to distributing the questionnaire, the following steps were undertaken: a) The original English instrument is translated into Indonesian; b) An English expert translated it back to English, allowing the researcher to compare it with the original and ensure consistency in meaning.

At the beginning of the digital survey (Google Form), participants were provided with a clear explanation regarding the study’s objectives, the confidential nature of the data, and how the results would be used. They were informed that their participation was entirely voluntary and that they could withdraw at any point. By proceeding to complete and submit the questionnaire, participants provided their implied informed consent. This study was conducted in accordance with the ethical principles of the Declaration of Helsinki. Given the non-invasive nature of the research and the use of a fully anonymous self-administered questionnaire, no personally identifiable information was collected from the respondents. According to the Ethical Guidelines for Research of Brawijaya University (available at: https://lppm.ub.ac.id/wp-content/uploads/PANDUAN-ETIKA-RISET2018-fix.pdf), formal ethical approval is not required for research involving minimal risk and total anonymity. Prior to participation, all respondents were provided with clear information regarding the study’s purpose and their right to withdraw, and their voluntary completion of the survey was considered as implied informed consent.

Informed consent was obtained from all participants prior to the data collection process. Given the sensitive nature of the study, which explores employee mental health and digital anxiety, written informed consent was intentionally waived to ensure the complete anonymity of the respondents. For questionnaires administered in person, verbal consent was obtained after participants were briefed on the study’s objectives, data confidentiality, and the voluntary nature of their involvement. Participation was considered as implied informed consent, evidenced by the voluntary completion and submission of the questionnaire. This approach was chosen to ensure that no physical signatures could link individual identities to their survey responses, thereby encouraging honest and unbiased participation.

A pilot test involving at least 30 questionnaires was conducted to assess the validity and reliability of the instrument, targeting postgraduate students from the Faculty of Economics and Business at Universitas Brawijaya. Data analysis for hypothesis testing utilized Partial Least Squares (PLS) via SmartPLS version 4.0 M3. PLS, a variance-based Structural Equation Modeling (SEM) method, is suitable for addressing issues like small sample sizes, missing data, and multicollinearity. The advantages of PLS include its applicability in theory development, capability to model multiple dependent and independent variables, and robustness against non-normal data.

The Partial Least Square (PLS) approach produces path coefficient values or statistical t values. For the implementation of the t test, the testing criteria for all hypothesis if the t-statistic value is greater than 1.64 then H0 is rejected, and the research hypothesis is supported.

The constructs in this study include digital training, role clarity, teamwork, transformational leadership, trends, regulatory support, ICT skills, and digital anxiety. Measurement instruments for these constructs are adapted from previous studies ( Akrong et al., 2022; AlBar & Hoque, 2019; Firk et al., 2023), enhancing the validity and reliability of the measurements. Each variable is assessed using a Likert scale from 1 to 5, where (1) Strongly Disagree, (2) Disagree, (3) Neutral, (4) Agree, and (5) Strongly Agree.

Before distributing the questionnaire, a pilot test was conducted to ensure the clarity and adequacy of the items. This pilot test involved 30 postgraduate students from the Faculty of Economics and Business at Brawijaya University. The results confirmed that the questionnaire items were both valid and reliable. Following this validation, the questionnaires were distributed to actual respondents in the field.

## 4. Results

A total of 283 questionnaires’ were received, of which 13 were excluded for not meeting the respondent criteria, specifically for not using digital technology in their work. Thus, 270 questionnaires were deemed valid for analysis. The majority of respondents were male (64.44%) compared to female (35.56%). In terms of position, most respondents held roles as department heads (48.89%) and supervisors (47.04%), indicating a predominance of middle-level management, which enhances the relevance of this study to managerial perspectives.

The results of the Total Effects test (path coefficients and t-values) for the main structural model are presented in Table 1. This study employs one-tailed hypothesis testing, with a critical value of 1.645 at a 5% significance level. Based on Table 1, hypotheses H1a, H1b, H1c, H1d, H2, H3a, and H3c are supported. A negative coefficient value indicates a negative effect on digital anxiety.

**Table 1.** Results of hypothesis testing.

Hypothesis	Construct	Coefficient	T value	P value	Decision
H1a	DT ➔ DA	-0,151	2,227	0,013 *	Supported
H1b	RC ➔ DA	-0,173	2,071	0,019 *	Supported
H1c	TS ➔ DA	-0,221	4,349	0,000 *	Supported
H1d	TL ➔ DA	-0,144	2,205	0,014 *	Supported
H2	RS ➔ DA	-0,105	2,476	0,007 *	Supported
H3a	ICTS*DT ➔ DA	-0,122	1,946	0,026 *	Supported
H3b	ICTS*RC ➔ DA	0,094	1,371	0,086	Not Supported
H3c	ICTS*TS ➔ DA	-0,099	1,885	0,030 *	Supported
H3d	ICTS*TL ➔ DA	0,014	0,242	0,404	Not Supported

* Indicates that the relationship is statistically significant and the hypothesis is supported (p < 0.05).

## 5. Discussion

This study investigates factors that mitigate digital anxiety among employees using digital technology in the finance departments of SOEs in Indonesia. The findings confirm that:

First, increased and improved digital training reduces digital anxiety, consistent with prior research ( Almajali et al., 2016; Firk et al., 2023; Rabaa, 2009). This aligns with social cognitive theory, which emphasizes the importance of observational learning in acquiring new skills ( Wood & Bandura, 1989). Regular training helps users develop skills and confidence, improving their cognitive and social abilities ( Akrong et al., 2022). As users become more proficient, they experience greater satisfaction and flexibility in using technology, leading to reduced anxiety. Moreover, effective training provides users with clear support channels, further alleviating technology-related anxiety. Empirical evidence suggests that individuals are more confident when they receive quality digital training from their organizations. Therefore, SOEs management should prioritize providing comprehensive and effective digital training to enhance employees’ capabilities in utilizing digital technologies, ultimately supporting overall performance.

Second, higher employee understanding of their work processes correlates with lower digital anxiety, as supported by previous research ( Adil et al., n.d.; Akrong et al., 2022; Samie et al., 2015). This aligns with social cognitive theory, which posits that self-confidence increases when individuals receive clear goals and effective information about their roles ( Wood & Bandura, 1989). When employees understand their responsibilities, they tend to feel more capable and self-efficacious ( Samie et al., 2015). This suggests that clarity in roles enhances technology usage and confidence. Therefore, SOEs management should provide clear job descriptions to prevent confusion among employees.

Third, improved teamwork is associated with reduced digital anxiety, consistent with findings from prior studies ( Attaran et al., 2019; Caesens et al., 2016; Sanyal & Hisam, 2018). Social cognitive theory states that self-confidence grows through support from colleagues ( Wood & Bandura, 1989). Collaborative environments foster motivation and enable employees to learn from one another. Companies should encourage teamwork and recognize employee contributions, as this support enhances confidence in using technology. SOEs management must ensure that employees can quickly access help from colleagues and IT departments.

Fourth, increased leader motivation for digital transformation leads to lower digital anxiety. This finding aligns with previous studies ( Hannah et al., 2016; Krishnan, 2005; Money, 2017). According to social cognitive theory, a leader’s self-efficacy is crucial for inspiring followers in dynamic environments ( Wood & Bandura, 1989). Leaders with high self-efficacy tend to be more able to inspire and motivate their followers. Transformational leaders can motivate employees to direct employees towards a specific digital transformation vision, for example the potential for digitalization in the finance department. Thus, employees who are motivated to carry out digital transformation tend not to feel anxiety. This empirical evidence has implications that individuals tend not to feel excessive anxiety if they have a leader who can motivate them towards a digital transformation vision.

Fifth, clearer government regulations regarding technology use correlate with lower digital anxiety. This finding is supported by earlier research ( Ali Raza, 2015; Hai & Kazmi, 2015; Mohamed Al Haderi, 2014; Reni & Ahmad, 2016; Tan & Teo, 1998, 2000). he theory of planned behavior suggests that individuals are more likely to use technology confidently when supported by external norms ( Ajzen, 1991). Support from other parties can be in the form of regulatory support from the government. Government support in terms of regulatory frameworks, security and privacy laws allow employees to develop better attitudes and confidence when using digital technology. The government is seen as an effective supporter, so it will influence people’s perceptions and behavior. This empirical evidence has implications that individuals tend to use digital technology with confidence if they feel they have government support in the form of regulations and laws. Thus, the government must emphasize and protect data security for technology users, provide support by providing incentives to users who use digital technology.

Sixth, digital training reduces digital anxiety, particularly when employees possess strong ICT skills. This finding aligns with previous studies ( Akrong et al., 2022; AlBar & Hoque, 2019). The Technology Acceptance Model (TAM) further elucidates this connection, positing that the perceived ease of use of technology is closely tied to a user’s skill level in operating it ( Ajzen, 1991). Employees who possess robust ICT skills are more likely to experience a sense of ease and proficiency when engaging with digital tools, thereby reducing anxiety associated with their use. Moreover, incorporating digital training not only equips employees with essential skills but also fosters a supportive environment that encourages adaptability to technological advancements. As a result, organizations that prioritize digital training can expect to see improved employee well-being and productivity, ultimately contributing to a more effective workforce.

Seventh, The study found that information and communication technology (ICT) skills do not serve as a moderating variable in the relationship between role clarity and digital anxiety. This contradicts findings by ( Qomariyah, 2016), who identified a significant moderating effect of ICT skills on the relationship between role clarity and individual motivation. The lack of support for this hypothesis (H3b) may be attributed to the demographic characteristics of the respondents, with 96.67% having over ten years of work experience. This extensive experience likely equips individuals with a strong understanding of their job roles, thereby enhancing their ability to utilize technology effectively, independent of their ICT skills ( Submitter et al., 2021). Long work experience fosters practical skill mastery and contextual understanding of digital technology use. Consequently, individuals with substantial experience can adapt to technological changes and operate efficiently in digital environments without relying heavily on formal ICT training.

Eighth, Strong teamwor supported by robust ICT skills, effectively reduces digital anxiety. Individuals with advanced ICT skills are better equipped to manage digital anxiety due to their ability to collaborate efficiently in technology-driven environments. This finding highlights that employees with higher ICT proficiency can amplify the positive effects of digital training and teamwork on alleviating digital anxiety. To address this issue, the Ministry of SOEs should prioritize enhancing employees’ ICT skills. By fostering a culture of continuous learning and collaboration, organizations can reduce digital anxiety and improve overall workforce adaptability in an increasingly digital workplace.

Ninth, ICT skills do not serve as a moderating variable in the relationship between transformational leadership and digital anxiety. This finding underscores the significance of organizational context in leadership and technology dynamics. In organizations led by transformational leaders, there is a strong emphasis on technology development, often facilitated by dedicated ICT departments. These departments provide specialized expertise, rendering individual ICT skills less critical in strategic decision-making. Transformational leaders excel at motivating team members to utilize technology effectively, independent of their ICT proficiency. As noted by Erman and Winario (2024), such leaders inspire innovation and foster an adaptive work culture. Thus, transformational leadership prioritizes creating an environment that promotes creativity and collaboration over mere technical mastery.

## 6. Conclusion

This study empirically demonstrates that organizational climate factors—namely digital training, role clarity, teamwork, and transformational leadership—along with regulatory support, negatively impact digital anxiety. Specifically, digital training reduces anxiety as employees interact more frequently with technology. Role clarity alleviates anxiety by ensuring employees understand their responsibilities. Teamwork diminishes anxiety through the support and appreciation employees receive from their colleagues. Transformational leadership fosters confidence in technology use by providing essential support. Additionally, regulatory support enhances employee attitudes and confidence when using digital technology through robust frameworks and privacy laws. The findings also indicate that information and communication technology (ICT) skills amplify the negative effects of digital training and teamwork on digital anxiety. Employees with stronger ICT skills are better equipped to leverage these factors to reduce anxiety.

This study has several limitations. First, the use of questionnaires may not capture emotional nuances or non-verbal reactions from respondents, which are crucial for understanding their responses. Future research could incorporate interviews to gain deeper insights into digital anxiety. Second, many respondents did not complete the open-ended questions in the Google Form survey. Future studies might consider placing open-ended questions at the beginning of the questionnaire to maintain respondent focus and enthusiasm.

## Data Availability

Zenodo: Dataset for Digitalization and Employee Mental Health in Indonesian SOEs: Mitigating Digital Anxiety in Finance Functions. https://doi.org/10.5281/zenodo.18242106 ( Hardanti et al., 2026) Zenodo: Survey Questionnaire for Digitalization and Employee Mental Health in Indonesian SOEs. https://doi.org/10.5281/zenodo.18242106 ( Hardanti et al., 2026) Data are available under the terms of the Creative Commons Attribution 4.0 International license (CC-BY 4.0).
