# Digitalization of Indonesian State-Owned Enterprises (SOEs)  and Employee Mental Health: Mitigating Digital Anxiety in Finance Functions

**DOI:** 10.12688/f1000research.176773.2

**Published:** 2026-04-08

**Authors:** Kurniasari Novi Hardanti, Sutrisno T, Erwin Saraswati, Arum Prastiwi

**Affiliations:** 1 Department of Accounting, Faculty of Economics and Business, Universitas Brawijaya, Malang, East Java, 65145, Indonesia

**Keywords:** Digital anxiety, organizational climate, regulatory support, and ICT skill

## Abstract

This study investigates factors that mitigate digital anxiety, focusing on organizational climate (digital training, role clarity, teamwork, transformational leadership), regulatory support, and information and communication technology (ICT) skills. Grounded in the Technology-Organization-Environment framework, this research provides a novel contribution by exploring these relationships in the context of state-owned enterprises (SOEs) in Indonesia. Data were collected through a survey targeting finance department employees in SOEs who utilize digital technology in their daily tasks. A total of 270 valid responses were analyzed using SmartPLS. The findings reveal that organizational climate variables along with regulatory support, significantly reduce digital anxiety. Moreover, ICT skills enhance the negative impact of digital training and teamwork on digital anxiety. However, the moderating role of ICT skills in strengthening the effects of role clarity and transformational leadership on digital anxiety was not supported. The practical implications of this study are significant for SOE management. To foster a supportive organizational climate, managers should ensure clearly defined roles through detailed job descriptions and encourage collaborative teamwork. Such measures can help employees address challenges collectively, thereby alleviating digital anxiety. These insights contribute to both academic literature on workplace digital transformation and managerial strategies for employee well-being.

## 1. Introduction

The Ministry of Communication and Informatics of Indonesia released the 2022 digital society index at 37.8 on a scale of 1-100, indicating significant challenges in digital competence among the population. A low IMD score is associated with heightened anxiety regarding the use of digital technologies, which can disrupt daily activities and overall well-being. From a self-regulation perspective, anxiety manifests as feelings of restlessness, worry, or fear ( Wood & Bandura, 1989).

Technological advancements contribute to this anxiety, as they are perceived to mimic human cognitive processes and threaten job security; projections suggest that between 400 to 800 million workers may be displaced by technology by 2030 ( Li & Huang, 2020). While digitalization offers numerous benefits, it also introduces risks that can trigger negative emotional responses such as anxiety ( Pfaffinger et al., 2021). Individuals may respond to such anxiety by either withdrawing or increasing their engagement with technology, a reaction influenced by their adaptive capabilities ( Carver & Scheier, 2021). Despite the pressing nature of digital anxiety as a social issue resulting from digital transformation, it remains underexplored in academic literature.

Digital anxiety represents a significant emotional challenge within corporate environments, providing insights into individual behaviors ( Ashkanasy & Daus, 2002; Bharat, 2011; Repenning et al., 2022). In the financial sector, this form of anxiety arises from the pressures of digital transformation involving AI, IoT, big data analytics, and ICT applications—tools that can enhance financial management but also exacerbate employee fears regarding job security ( Firk et al., 2023; Nugroho, 2019). The presence of digital anxiety can diminish both individual and organizational performance ( Bala & Venkatesh, 2016; Kent et al., 2023; Pfaffinger et al., 2021), leading employees to perceive digitalization as a threat rather than an opportunity for enhancing work quality ( Firk et al., 2023). This emotional state can result in frustration, burnout, loss of motivation, and ultimately disengagement from work processes. Ultimately digital anxiety can be a barrier to digital transformation in finance, but it is still unclear about the level of anxiety in the finance function and how to reduce digital anxiety in employees.

To address these challenges, it is crucial to enhance individual self-efficacy in navigating technology through three key mechanisms: competence development, fostering belief in one’s ability to manage situations, and increasing motivation for specific behaviors ( Wood & Bandura, 1989). This study aims to investigate how these mechanisms interact with four critical organizational climate factors: digital training effectiveness, role clarity, teamwork dynamics, and transformational leadership. Organizational climate is believed to have a direct impact on employee motivation in using a technology ( Madhukar et al., 2017). The implementation of digital transformation is highly dependent on the organizational climate ( Rücker et al., 2021).

This study investigates environmental factors that potentially alleviate digital anxiety, framed within the Technology-Organization-Environment (TOE) model. The TOE framework, introduced by Tornatzky and Fleischer (1990), emphasizes that technological, organizational, and environmental elements significantly impact technology adoption by both organizations and individuals. Environmental factors are crucial for comprehending technology usage ( AlBar & Hoque, 2019). Furthermore, the Theory of Planned Behavior (TPB) effectively elucidates how external environmental aspects influence technology utilization ( Tan & Teo, 2000). The proliferation of technology is facilitated when the requisite technological infrastructure is accessible and user-friendly. Government intervention and leadership are pivotal in promoting innovation diffusion among individuals ( Hai & Kazmi, 2015; Tan & Teo, 1998).

Within the framework of the Technology Acceptance Model (TAM), technological factors play a critical role in user acceptance of information technology ( Davis et al., 1989). TAM effectively addresses how technological aspects can diminish digital anxiety ( Akrong et al., 2022; Gefen & Straub, 2004). The perceived ease of use within TAM correlates closely with users’ ICT (Information and Communication Technology) skills; users expect technology to be intuitive, reflecting their foundational skills.

ICT has revolutionized various life aspects ( Bosamia, 2013; Jandsalar, 2022). However, a significant barrier to successful digital transformation is the scarcity of human resources skilled in ICT. The influence of ICT proficiency on digital anxiety is paramount during digitalization efforts. Extended computer usage enhances user behavior concerning ERP systems in Ghana indicating that increased experience with technology fosters improved engagement ( Akrong et al., 2022). Camilleri (2020) found the same thing, that skills in using a technology will increase user involvement in using e-government systems.

This study addresses a notable gap in research concerning digital anxiety ( Firk et al., 2023) *.* Digital anxiety poses challenges to financial transformation; however, the specific levels of anxiety within finance functions and strategies for alleviating employee concerns remain underexplored. The ongoing digital transformation within SOEs has garnered attention as these entities increasingly leverage digital technologies for operational efficiency and expedited decision-making. State-owned enterprises in Indonesia has adopted blockchain technology across various facets of digital finance—including trade finance, carbon transactions, and public financial management—facilitating secure data exchanges that are anticipated to boost transaction volumes and corporate revenues.

While digital anxiety is a global phenomenon, its manifestation in emerging economies with strong state-led digital mandates, such as Indonesia, remains under-researched. This study offers a unique perspective by focusing on Indonesian State-Owned Enterprises (SOEs), which serve as the backbone of the national economy. The Indonesian context provides a unique selling point as it demonstrates how centralized regulatory frameworks in a developing nation act as a critical buffer against technostress, offering a benchmark for other Global South countries undergoing similar digital transformations.

The objectives of this study are threefold: to examine the impact of organizational climate—specifically digital training, role clarity, teamwork, and transformational leadership—on digital anxiety; to assess the influence of regulatory support on digital anxiety; and to evaluate the moderating effect of ICT skills on the relationship between organizational climate factors and digital anxiety.

This study aims to refine existing theories by integrating social cognitive theory, Technology-Organization-Environment, TAM, and TPB into a single model. Practically, the findings serve as valuable insights for systems analysts and organizational management. For IT analysts tasked with implementing information systems within finance departments, it is essential to consider how information technology can enhance job efficiency, accelerate processes, boost productivity, and elevate employee performance. For organizational leaders, this study offers guidance on fostering a positive organizational climate conducive to effective digital transformation initiatives.

## 2. Literature review and hypothesis development

### 2.1 Research model and hypothesis

**Figure 1.**
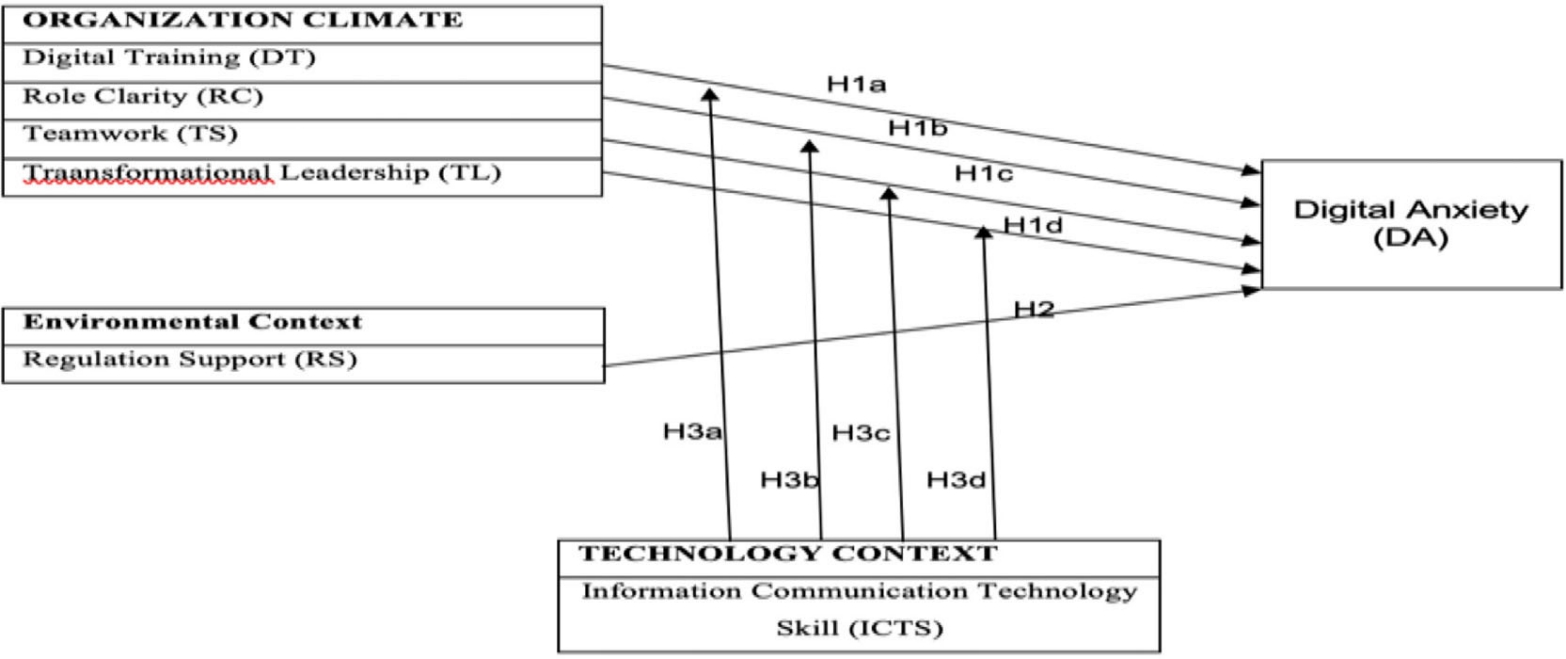
Proposed theoretical framework.

Figure 1 shows the research model developed from the previously described literature. The model in this study seeks to understand the factors that can mitigate digital anxiety. This study develops a social cognitive theory in organizations built by ( Wood & Bandura, 1989). This theory posits that an individual’s self-efficacy is influenced by the surrounding organizational factors. In addition to organizational influences, the Technology-Organization-Environment (TOE) model highlights the importance of environmental and technological factors ( Tornatzky & Fleischer, 1990).

*2.1.1 Digital training and digital anxiety*

Competency development is essential for organizational change, aligning with social cognitive theory ( Wood & Bandura, 1989). This theory posits that enhancing competencies within companies can improve understanding of technology usage determinants. Goretzki et al., (2013) emphasize that competency development is crucial for organizational transformation, particularly through digital training. Regular digital training is expected to reduce employees’ digital anxiety.

Training can help employees adapt to change and be positive about the digital transformation that is happening in the finance department. Based on these insights, the following hypothesis is proposed:

H1a: Digital training negatively affects digital anxiety.

*2.1.2 Role clarity hypothesis on digital anxiety*

The second mechanism in social cognitive theory emphasizes the importance of individuals’ belief in their ability to cope with situations ( Wood & Bandura, 1989). This confidence is crucial for self-regulation and emotional responses ( Firk et al., 2023). Beliefs are shaped by the organizational context, as outlined in social cognitive theory ( Wood & Bandura, 1989). Clear role definitions during digital transformation enhance individual confidence. Role clarity is essential for successful technology implementation, as it involves understanding organizational expectations regarding job outcomes (Y. Li et al., 2020). Research indicates that role clarity is linked to higher job satisfaction (J. Li & Huang, 2020; Thangavelu & Sudhahar, 2017). Employees with well-defined goals and responsibilities are more likely to know whom to approach for support and guidance in using new systems. This clarity significantly impacts individual performance. Furthermore, when employees clearly understand their digital responsibilities, it reduces the cognitive load associated with technological changes. Role clarity acts as a psychological safety net, ensuring that digitalization is perceived as a structural improvement rather than a source of job ambiguity. Based on this rationale, the following hypothesis is proposed:

H1b: Role clarity negatively affects digital anxiety.

*2.1.3 Teamwork hypothesis on digital anxiety*

The second mechanism in Social Cognitive Theory relates to the belief that individuals must be able to cope with situations ( Wood & Bandura, 1989). Confidence in managing specific situations is critical for self-regulation and emotional responses ( Firk et al., 2023) and is influenced by the surrounding organizational context ( Wood & Bandura, 1989).

Teamwork plays a crucial role in supporting individuals during digital transformation. It refers to the ability of technology users to receive timely assistance from colleagues, service providers, and the information systems department ( Akrong et al., 2022). Teamwork is fostered through collaboration, mutual support, and shared accountability ( Caesens et al., 2016; Hanaysha & Tahir, 2016). Research demonstrates that teamwork significantly enhances the adoption of information systems ( Costa et al., 2020) and underscores its importance in ERP system implementation ( Almajali et al., 2016). Based on this rationale, the following hypothesis is proposed:

H1c: Teamwork negatively affects digital anxiety.

*2.1.4 Transformational leadership hypothesis on digital anxiety*

The third mechanism in Social Cognitive Theory pertains to employee motivation to achieve specific goals ( Wood & Bandura, 1989). Motivated employees are more likely to engage in required behaviors, thus reducing susceptibility to negative emotions ( Bandura, 1988). Goretzki et al., (2013) highlight that leadership actions—such as storytelling, effective communication, and leading by example—can foster motivation and facilitate organizational change. Transformational leaders can effectively motivate employees to embrace the digital transformation vision, especially in the financially strategic sector, ultimately alleviating digital anxiety. Transformational leadership is inherently about change ( Money, 2017). Transformational leaders can encourage their followers to change their expectations, perceptions, and motivations to work toward common goals. Based on this analysis, the following hypothesis is proposed:

H1d: Transformational leadership negatively affects digital anxiety.

*2.1.5 Hypothesis of regulatory support for digital anxiety*

Regulatory support refers to the policies and regulations that facilitate technology adoption, enhancing the competitiveness of business processes ( Pudjianto et al., 2011). Numerous studies have identified government regulatory support as a key driver of technology adoption ( Amini et al., 2014; Charag et al., 2020; W. C. Chen et al., 2019; Hai & Kazmi, 2015). Baker (2011) indicates that environmental factors positively influence usage behavior, suggesting that greater government support correlates with higher technology adoption rates. The influence of government support formulated and proven by ( Rogers, 1983) in TOE, has been supported by many studies ( AlBar & Hoque, 2019; Junior et al., 2019). Junior et al., (2019) conducted a study of 375 farmers in Brazil who use ERP in their work processes. Based on this analysis, the following hypothesis is proposed:

H2: Regulatory support negatively affects digital anxiety.

*2.1.6 ICT Skills as a moderating variable*

This study proposes that ICT (Information and Communication Technology) skills will moderate the influence between independent variable and dependent variable. Derived from the Technology Acceptance Model ( Davis et al. 1989), ICT skills are crucial for reducing digital anxiety by enhancing perceived ease of use. Proficient ICT skills enable users to navigate technology more comfortably, which is essential for successful digital transformation ( Marston et al., 2011). Research indicates that inadequate ICT skills can lead to decreased motivation and increased anxiety among users ( Lutovac & Manojlov, 2012). Akrong et al., (2022) found that prolonged computer use positively influences system usage behavior. As individuals become more adept at using technology, their self-confidence grows, thereby alleviating anxiety ( Alkhawaja et al., 2021; Iraola-Real et al., 2023). In the context of Indonesian SOEs, regulatory support provides a formal mandate that legitimizes the digital transition. Clear government guidelines offer a sense of stability, signaling to employees that the transformation is part of a national roadmap, thereby reducing individual fears of systemic failure or job insecurity during the process. Thus, the following hypothesis is proposed:

H3a: ICT skills strengthen the negative effect of digital training on digital anxiety.

H3b: ICT skills strengthen the negative influence of role clarity on digital anxiety.

H3c: ICT skills strengthen the negative effect of teamwork on digital anxiety.

H3d: ICT skills strengthen the negative influence of transformational leadership on digital anxiety.

## 3. Methods

### 3.1 Research design and approach

This study adopts a quantitative explanatory research design to investigate the factors mitigating digital anxiety among employees in Indonesian State-Owned Enterprises (SOEs). The research utilizes a cross-sectional survey approach, integrating the Technology-Organization-Environment (TOE) framework, Social Cognitive Theory (SCT), and the Technology Acceptance Model (TAM) to provide a holistic analysis of organizational and environmental influences.

### 3.2 Population and sampling

The population of this study consists of individuals working at State-Owned Enterprises (SOEs) in Indonesia, specifically those within departments undergoing digital transformation, such as finance, accounting, internal audit, and taxation. These departments were selected due to the high-stakes nature of their digital transition.

A purposive sampling method was employed to select respondents based on specific criteria: (1) active employment in an Indonesian SOE, and (2) involvement in finance-related functions. The final sample comprised 270 respondents. Notably, the majority of respondents (96.67%) possessed over 10 years of professional experience, reflecting the senior leadership and established workforce dominant in the finance departments of Indonesian SOEs during this digital transition period.

### 3.3 Measurement of variables

The constructs in this study were adapted from established literature to ensure content validity. Measurement instruments for digital training, role clarity, teamwork, transformational leadership, trends, regulatory support, ICT skills, and digital anxiety were adapted from previous studies ( Akrong et al., 2022; AlBar & Hoque, 2019; Firk et al., 2023). Each variable was assessed using a five-point Likert scale ranging from (1) “Strongly Disagree” to (5) “Strongly Agree”.

### 3.4 Data collection procedure

Data collection was conducted between 2024-2025. To ensure instrument validity, the original English instrument underwent a rigorous translation and back-translation process by independent language experts.

The survey was administered electronically via Google Forms, resulting in 270 valid online responses. The distribution of the survey link followed a hybrid outreach strategy: the majority of the links were distributed through professional networks and official email/messaging channels, while a portion was shared in person during site visits to the finance departments of various SOEs. This in-person approach allowed the researcher to provide a verbal briefing regarding the study’s objectives and ensure participants’ understanding before they accessed the digital form. On average, respondents took approximately 10–15 minutes to complete the questionnaire.

### 3.5 Ethical considerations and informed consent

This study was conducted in accordance with the ethical principles of the Declaration of Helsinki and the Ethical Guidelines for Research of Universitas Brawijaya. Given the non-invasive nature of the study and total anonymity, formal ethical approval was not required. Written informed consent was intentionally waived to maintain complete respondent anonymity, particularly given the sensitive nature of mental health and anxiety. Instead, implied informed consent was obtained, evidenced by the voluntary completion and submission of the questionnaire after participants were fully briefed on their rights to withdraw at any time.

### 3.6 Pilot test

Prior to the full-scale distribution, a pilot test was conducted involving 30 postgraduate students from the Faculty of Economics and Business at Universitas Brawijaya. This step ensured the clarity and adequacy of the items. The results confirmed that the instrument was both valid and reliable. Data from this pilot test were used solely for instrument validation and were not included in the final 270-sample data analysis.

### 3.7 Data analysis

Hypothesis testing was performed using Structural Equation Modeling (SEM) via Partial Least Squares (PLS) with SmartPLS version 4.0 M3. PLS-SEM was chosen for its robustness in handling non-normal data, its suitability for theory development, and its capability to model multiple complex relationships simultaneously. The significance of the path coefficients was tested using the bootstrapping method. Testing criteria followed a t-statistic threshold of >1.64 (one-tailed test) to determine if the research hypothesis were supported.

## 4. Results

A total of 283 questionnaires’ were received, of which 13 were excluded for not meeting the respondent criteria, specifically for not using digital technology in their work. Thus, 270 questionnaires were deemed valid for analysis. The majority of respondents were male (64.44%) compared to female (35.56%). In terms of position, most respondents held roles as department heads (48.89%) and supervisors (47.04%), indicating a predominance of middle-level management, which enhances the relevance of this study to managerial perspectives.

The measurement model was evaluated to ensure the reliability and validity of the constructs. As shown in Table 1, all Cronbach’s Alpha and Composite Reliability (CR) for all constructs were above 0.70, indicating high internal consistency. The Average Variance Extracted (AVE) for each construct also surpassed 0.50, confirming adequate convergent validity.

**Table 1.** Measurement model result.

Konstruk	AVE	*Composite Reliability*	*Cronbachs Alpha*
Digital Training (DT)	0.819	0.932	0.890
Role Clarity (RC)	0.838	0.954	0.935
Teamwork & Support (TS)	0.843	0.955	0.938
Transformational Leadership (TL)	0.798	0.959	0.949
Regulation Support (RS)	0.877	0.934	0.859
ICT Skill (ICTS)	0.916	0.970	0.954
Digital Anxiety (DA)	0.863	0.969	0.960

To assess discriminant validity, the Fornell-Larcker criterion was employed. As shown in Table 2, the square root of the Average Variance Extracted (AVE) for each construct represented by the diagonal elements in bold exceeds the correlations between that construct and any other constructs in the model. This demonstrates that each construct is statistically distinct and shares more variance with its assigned indicators than with other variables, thereby confirming adequate discriminant validity for the measurement model.

**Table 2.** Discriminant validity (Fornell-Larcker criterion).

Construct	DA	DT	ICTS	RC	RS	TS	TL
DA	0.929						
DT	-0.713	0.905					
ICTS	0.423	-0.073	0.957				
RC	-0.682	0.806	-0.057	0.915			
RS	-0.647	0.741	-0.043	0.697	0.936		
TS	-0.748	0.730	-0.259	0.665	0.625	0.918	
TL	-0.665	0.791	0.008	0.780	0.729	0.680	0.893

The results of the Total Effects test (path coefficients and t-values) for the main structural model are presented in Table 3. This study employs one-tailed hypothesis testing, with a critical value of 1.645 at a 5% significance level. Based on Table 3, hypotheses H1a, H1b, H1c, H1d, H2, H3a, and H3c are supported. A negative coefficient value indicates a negative effect on digital anxiety.

**Table 3.** Results of hypothesis testing.

Hypothesis	Construct	Coefficient	T value	P value	Decision
H1a	DT ➔ DA	-0.151	2.227	0.013 *	Supported
H1b	RC ➔ DA	-0.173	2.071	0.019 *	Supported
H1c	TS ➔ DA	-0.221	4.349	0.000 *	Supported
H1d	TL ➔ DA	-0.144	2.205	0.014 *	Supported
H2	RS ➔ DA	-0.105	2.476	0.007 *	Supported
H3a	ICTS*DT ➔ DA	-0.122	1.946	0.026 *	Supported
H3b	ICTS*RC ➔ DA	0.094	1.371	0.086	Not Supported
H3c	ICTS*TS ➔ DA	-0.099	1.885	0.030 *	Supported
H3d	ICTS*TL ➔ DA	0.014	0.242	0.404	Not Supported

* Indicates that the relationship is statistically significant and the hypothesis is supported (p < 0.05).

## 5. Discussion

This study investigates factors that mitigate digital anxiety among employees using digital technology in the finance departments of SOEs in Indonesia. The findings confirm that:

First, increased and improved digital training reduces digital anxiety, consistent with prior research ( Almajali et al., 2016; Firk et al., 2023; Rabaa, 2009). This aligns with social cognitive theory, which emphasizes the importance of observational learning in acquiring new skills ( Wood & Bandura, 1989). Regular training helps users develop skills and confidence, improving their cognitive and social abilities ( Akrong et al., 2022). As users become more proficient, they experience greater satisfaction and flexibility in using technology, leading to reduced anxiety. Moreover, effective training provides users with clear support channels, further alleviating technology-related anxiety. Empirical evidence suggests that individuals are more confident when they receive quality digital training from their organizations. Therefore, SOEs management should prioritize providing comprehensive and effective digital training to enhance employees’ capabilities in utilizing digital technologies, ultimately supporting overall performance.

Second, higher employee understanding of their work processes correlates with lower digital anxiety, as supported by previous research ( Adil et al., n.d.; Akrong et al., 2022; Samie et al., 2015). This aligns with social cognitive theory, which posits that self-confidence increases when individuals receive clear goals and effective information about their roles ( Wood & Bandura, 1989). When employees understand their responsibilities, they tend to feel more capable and self-efficacious ( Samie et al., 2015). This suggests that clarity in roles enhances technology usage and confidence. Therefore, SOEs management should provide clear job descriptions to prevent confusion among employees.

Third, improved teamwork is associated with reduced digital anxiety, consistent with findings from prior studies ( Attaran et al., 2019; Caesens et al., 2016; Sanyal & Hisam, 2018). Social cognitive theory states that self-confidence grows through support from colleagues ( Wood & Bandura, 1989). Collaborative environments foster motivation and enable employees to learn from one another. Companies should encourage teamwork and recognize employee contributions, as this support enhances confidence in using technology. SOEs management must ensure that employees can quickly access help from colleagues and IT departments.

Fourth, increased leader motivation for digital transformation leads to lower digital anxiety. This finding aligns with previous studies ( Hannah et al., 2016; Krishnan, 2005; Money, 2017). According to social cognitive theory, a leader’s self-efficacy is crucial for inspiring followers in dynamic environments ( Wood & Bandura, 1989). Leaders with high self-efficacy tend to be more able to inspire and motivate their followers. Transformational leaders can motivate employees to direct employees towards a specific digital transformation vision, for example the potential for digitalization in the finance department. Thus, employees who are motivated to carry out digital transformation tend not to feel anxiety. This empirical evidence has implications that individuals tend not to feel excessive anxiety if they have a leader who can motivate them towards a digital transformation vision.

Fifth, clearer government regulations regarding technology use correlate with lower digital anxiety. This finding is supported by earlier research ( Ali Raza, 2015; Hai & Kazmi, 2015; Mohamed Al Haderi, 2014; Reni & Ahmad, 2016; Tan & Teo, 1998, 2000). he theory of planned behavior suggests that individuals are more likely to use technology confidently when supported by external norms ( Ajzen, 1991). Support from other parties can be in the form of regulatory support from the government. Government support in terms of regulatory frameworks, security and privacy laws allow employees to develop better attitudes and confidence when using digital technology. The government is seen as an effective supporter, so it will influence people’s perceptions and behavior. This empirical evidence has implications that individuals tend to use digital technology with confidence if they feel they have government support in the form of regulations and laws. While digital anxiety is a global phenomenon, its manifestation in emerging economies with strong state-led digital mandates, such as Indonesia, remains under-researched. This study offers a unique perspective by focusing on Indonesian State-Owned Enterprises (SOEs), which serve as the backbone of the national economy. The Indonesian context provides a Unique Selling Point (USP) as it demonstrates how centralized regulatory frameworks in a developing nation act as a critical buffer against technostress, offering a benchmark for other Global South countries undergoing similar digital transformations. Thus, the government must emphasize and protect data security for technology users, provide support by providing incentives to users who use digital technology.

Sixth, digital training reduces digital anxiety, particularly when employees possess strong ICT skills. This finding aligns with previous studies ( Akrong et al., 2022; AlBar & Hoque, 2019). The Technology Acceptance Model (TAM) further elucidates this connection, positing that the perceived ease of use of technology is closely tied to a user’s skill level in operating it ( Ajzen, 1991). Employees who possess robust ICT skills are more likely to experience a sense of ease and proficiency when engaging with digital tools, thereby reducing anxiety associated with their use. Moreover, incorporating digital training not only equips employees with essential skills but also fosters a supportive environment that encourages adaptability to technological advancements. As a result, organizations that prioritize digital training can expect to see improved employee well-being and productivity, ultimately contributing to a more effective workforce.

Seventh, The study found that information and communication technology (ICT) skills do not serve as a moderating variable in the relationship between role clarity and digital anxiety. This contradicts findings by ( Qomariyah, 2016), who identified a significant moderating effect of ICT skills on the relationship between role clarity and individual motivation. The lack of support for this hypothesis (H3b) may be attributed to the demographic characteristics of the respondents, with 96.67% having over ten years of work experience. This extensive experience likely equips individuals with a strong understanding of their job roles, thereby enhancing their ability to utilize technology effectively, independent of their ICT skills ( Submitter et al., 2021). Long work experience fosters practical skill mastery and contextual understanding of digital technology use. Consequently, individuals with substantial experience can adapt to technological changes and operate efficiently in digital environments without relying heavily on formal ICT training.

Eighth, Strong teamwor supported by robust ICT skills, effectively reduces digital anxiety. Individuals with advanced ICT skills are better equipped to manage digital anxiety due to their ability to collaborate efficiently in technology-driven environments. This finding highlights that employees with higher ICT proficiency can amplify the positive effects of digital training and teamwork on alleviating digital anxiety. To address this issue, the Ministry of SOEs should prioritize enhancing employees’ ICT skills. By fostering a culture of continuous learning and collaboration, organizations can reduce digital anxiety and improve overall workforce adaptability in an increasingly digital workplace.

Ninth, ICT skills do not serve as a moderating variable in the relationship between transformational leadership and digital anxiety. This finding underscores the significance of organizational context in leadership and technology dynamics. In organizations led by transformational leaders, there is a strong emphasis on technology development, often facilitated by dedicated ICT departments. These departments provide specialized expertise, rendering individual ICT skills less critical in strategic decision-making. Transformational leaders excel at motivating team members to utilize technology effectively, independent of their ICT proficiency. As noted by Erman and Winario (2024), such leaders inspire innovation and foster an adaptive work culture. Thus, transformational leadership prioritizes creating an environment that promotes creativity and collaboration over mere technical mastery.

## 6. Implication of the study

### 6.1 Theoretical implications

This study contributes to the literature on digital transformation and occupational health by integrating the TOE framework and SCT to explain digital anxiety in a specific public sector context. The findings highlight that in developing economies like Indonesia, Regulatory Support and Role Clarity serve as foundational pillars that mitigate the psychological stressors associated with technology adoption. This adds a new dimension to existing models by demonstrating that environmental and structural certainties are as critical as individual technical skills in reducing employee anxiety.

### 6.2 Practical and managerial implications

For practitioners and decision-makers in State-Owned Enterprises (SOEs), this research offers several actionable insights:

a. Management should prioritize clear communication regarding digital roles and responsibilities. Reducing job ambiguity during digital transitions significantly lowers the cognitive load on employees, particularly for the senior workforce.
b. Leadership should utilize government digital roadmaps not just as compliance requirements, but as a “stability signal” to reassure employees that the transformation is a structured, national-scale initiative.
c. Beyond technical ICT skills, training programs should incorporate “soft-skill” components that address the emotional and psychological aspects of technostress, fostering a more resilient digital culture within finance departments.
d. Transformational leaders must actively participate in the digital transition by providing a vision that emphasizes employee well-being alongside operational efficiency.

## 7. Conclusion

This study empirically demonstrates that organizational climate factors—namely digital training, role clarity, teamwork, and transformational leadership—along with regulatory support, negatively impact digital anxiety. Specifically, digital training reduces anxiety as employees interact more frequently with technology. Role clarity alleviates anxiety by ensuring employees understand their responsibilities. Teamwork diminishes anxiety through the support and appreciation employees receive from their colleagues. Transformational leadership fosters confidence in technology use by providing essential support. Additionally, regulatory support enhances employee attitudes and confidence when using digital technology through robust frameworks and privacy laws. The findings also indicate that information and communication technology (ICT) skills amplify the negative effects of digital training and teamwork on digital anxiety. Employees with stronger ICT skills are better equipped to leverage these factors to reduce anxiety. While the findings are grounded in the Indonesian regulatory context, they provide a valuable benchmark for other emerging economies undergoing state-led digital transformations, highlighting the universal importance of structured institutional support in mitigating technostress.

This study has several limitations. First, the use of questionnaires may not capture emotional nuances or non-verbal reactions from respondents, which are crucial for understanding their responses. Digital anxiety is a complex emotional state; thus, future research could incorporate in-depth interviews to gain a more personal understanding of how employees experience this emotional hurdle during digital transformation. Second, this study’s sample was predominantly composed of senior employees with over 10 years of experience. While this reflects the current professional landscape in Indonesian SOEs, future studies should ensure a broader distribution of age and tenure to investigate if digital native employees or new hires experience digital anxiety differently. Third, many respondents did not complete the open-ended questions in the Google Form survey. Future studies might consider placing open-ended questions at the beginning of the questionnaire to maintain respondent focus and enthusiasm.

## Data Availability

Zenodo: Dataset for Digitalization and Employee Mental Health in Indonesian SOEs: Mitigating Digital Anxiety in Finance Functions. https://doi.org/10.5281/zenodo.18242106 ( Hardanti et al., 2026) Zenodo: Survey Questionnaire for Digitalization and Employee Mental Health in Indonesian SOEs. https://doi.org/10.5281/zenodo.18242106 ( Hardanti et al., 2026) Data are available under the terms of the Creative Commons Attribution 4.0 International license (CC-BY 4.0).
