# Supplementary material for: Digitalization of Indonesian State-Owned Enterprises (SOEs)  and Employee Mental Health: Mitigating Digital Anxiety in Finance Functions
Source: F1000Res. 2026 Apr 8;15:282. Originally published 2026 Feb 17. [Version 2] doi: 10.12688/f1000research.176773.2 (PMC13250512; doi:10.12688/f1000research.176773.2)
Supplement: Peer review history

# Reviewer report: Reviewer response for version 2

**DOI:** 10.5256/f1000research.197977.r487968

**Published:** 2026-06-09

**Authors:** Mohammad Ichsan

**Affiliations:** 1 Binus Business School, Bina Nusantara University, Jakarta, Indonesia

In general, this article is very interesting, and the authors have used sufficient scientific steps in doing it.

 Introduction - this section lacks an explanation of the specific reason why this study is so important to be performed. The authors should explain both practical and research gaps, and show how this study can address them.

 Literature review - The theoretical aspects have to be explained in this section prior to building up the research model. Explain how the proposed theoretical perspectives can be used to solve the problem. Maybe it is an insignificant concern; I prefer to use ellipses instead of rectangles in the proposed research model.

 Method and tools - the authors need to define the minimum proposed samples to be used in the method. Furthermore, the final samples are supposed to be explained in the discussion section,  not in this section. The unit of analysis is also need to be explained. 

 Results - I would suggest the authors start with demographic data analysis prior to SEM analysis. I would also suggest that the HTMT method is used rather than Fornell Lacker. This justify (please see the paper's DOI as reference). Do also PLSPredict's analysis to ensure the model 'fit' of PLS analysis. Please also show the figure of structural model analysis result in this section. 

 Discussion - sufficient enough in highlighting of fulfullment of the research objectives.

 Conclusion - Typically, the study implication is part of the conclusion, but if it is not, that it's ok as this has been clearly explained.

Is the work clearly and accurately presented and does it cite the current literature?

Yes

If applicable, is the statistical analysis and its interpretation appropriate?

Partly

Are all the source data underlying the results available to ensure full reproducibility?

Yes

Is the study design appropriate and is the work technically sound?

Yes

Are the conclusions drawn adequately supported by the results?

Yes

Are sufficient details of methods and analysis provided to allow replication by others?

Yes

Reviewer Expertise:

decision science, project management, operations and supply chain management

I confirm that I have read this submission and believe that I have an appropriate level of expertise to confirm that it is of an acceptable scientific standard, however I have significant reservations, as outlined above.

## References

[1] : When to use and how to report the results of PLS-SEM. *European Business Review* .2019;31(1) : 10.1108/EBR-11-2018-0203 2-24 10.1108/EBR-11-2018-0203

# Reviewer report: Reviewer response for version 1

**DOI:** 10.5256/f1000research.194871.r459797

**Published:** 2026-03-10

**Authors:** Hazlina Binti Shaik Md Noor Alam

**Affiliations:** 1 Universiti Kebangsaan Malaysia, Selangor, Malaysia

This review evaluates the study titled "Factors that Mitigate Digital Anxiety," which explores the intersection of organizational climate, regulatory support, and ICT skills within the framework of the Technology-Organization-Environment (TOE) model.

 The research investigates how four organizational climate factors (digital training, role clarity, teamwork, and transformational leadership) and one environmental factor (regulatory support) influence digital anxiety among finance department employees in Indonesian SOEs. Utilizing a sample of 270 respondents and SmartPLS analysis, the study concludes that all primary factors significantly reduce digital anxiety. Furthermore, it identifies ICT skills as a moderator that strengthens the anxiety-reducing effects of training and teamwork, though it found no moderating effect on role clarity or leadership.

 Strong Theoretical Integration: The authors successfully synthesize multiple frameworks, including Social Cognitive Theory (SCT), the TOE framework, and the Technology Acceptance Model (TAM). This multi-lens approach provides a holistic view of both internal psychological states and external organizational pressures.

 Strengths

 Contextual Relevance: By focusing on the finance departments of Indonesian SOEs, the study addresses a specific "high-stakes" environment where digital transformation is rapid but often met with resistance due to job security fears.

 Practical Utility: The findings offer actionable frameworks for SOE managers, such as the necessity of combining technical training with clear job descriptions to manage employee well-being.

 Weaknesses

 Sample Bias & Demographics: A significant majority (96.67%) of respondents have over 10 years of experience.It limits the generalizability of the findings to younger, "digital native" employees or new hires who may experience digital anxiety differently.

 Self-Reporting Limitations: Digital anxiety is a sensitive psychological state. Relying solely on anonymous surveys may lead to social desirability bias, where employees underreport anxiety to appear more competent.

 Absence of Qualitative Depth: While the study mentions "qualitative insights," the discussion is primarily quantitative. The conclusion even notes that many respondents skipped open-ended questions, leaving a gap in understanding the nuance of how anxiety feels in this specific cultural context.

 Geographic Specificity: The reliance on Indonesian-specific regulatory support,makes it difficult to apply the "Regulatory Support" findings to countries with different legal infrastructures.

 Age-Diverse Sampling: Ensure a broader distribution of age and tenure to see if ICT skills play a more significant moderating role for junior employees compared to the veterans featured in this study.

 On the human/personnel element: The article rightly identifies that digital transformation is not just a technical hurdle but an emotional one. However, the study could have been strengthened by exploring more on digital anxiety.

Is the work clearly and accurately presented and does it cite the current literature?

Yes

If applicable, is the statistical analysis and its interpretation appropriate?

I cannot comment. A qualified statistician is required.

Are all the source data underlying the results available to ensure full reproducibility?

Yes

Is the study design appropriate and is the work technically sound?

Yes

Are the conclusions drawn adequately supported by the results?

Yes

Are sufficient details of methods and analysis provided to allow replication by others?

Yes

Reviewer Expertise:

Corporate Law,Contract Law,Media Law,Corporate Governance,Business Studies,Entrepreneurship,Environmental Law

I confirm that I have read this submission and believe that I have an appropriate level of expertise to confirm that it is of an acceptable scientific standard.

## References

[1] Influence of digital transformation on employee innovative behavior: roles of challenging appraisal, organizational culture support, and transformational leadership style authors.10.3389/fpsyg.2025.1532977PMC1196380840177047

[2] Digital transformation: A multidisciplinary reflection and research agenda authors.

[3] The Impact of Technostress on Role Stress and Productivity author.

[4] Anxiety in a digitalised work environment authors.

[5] technology adoption in digital era:the role of public policy authors.

[6] Factors Influencing Digital Technology Adoption and Use Among MSMEs in Indonesia authors.

[7] determinants of e government assimilation in Indonesia:an empirical investigation using a toe framwork authors:boni pudjianto,hangjung zo,Andrew p ciganek,jae jeung rho year.

[8] Perceived Usefulness, Perceived Ease of Use, and User Acceptance of Information Technology authors.

# Reviewer report: Reviewer response for version 1

**DOI:** 10.5256/f1000research.194871.r459802

**Published:** 2026-03-10

**Authors:** Godwin Banafo Akrong Akrong

**Affiliations:** 1 McGill University, Montreal, Canada

The author(s) have carried out an interesting study; however, I have some concerns:

1. In the title, the author(s) should provide the full text of the abbreviation before presenting it. E.g., SOE
2. Section 2 should be Literature Review and Hypothesis Development.
3. It would be important for the author(s) to briefly discuss the literature behind their work before proceeding to narrow down to the hypothesis development and their model.
4. Section 2.1.1 should be Digital training and digital anxiety. The author(s) do not need to restate the hypothesis.
5. The author(s) should strengthen the arguments that support their proposed hypothesis presented in Sections 2.1.2 and 2.1. 6.
6. Section 3 should be clearly organized into subsections to help readers understand how the author(s) conducted their study, as follows: a. Research design/approach; b. Population/location; c. Sampling; d. Measurement of the variables; e. Data collection procedure; f. Data analysis  
7. Section 3 should be labelled as Methods.
8. In Section 3, were the initial pilot test results added to the final data collected?
9. Was the questionnaire initially tested before distribution?
10. Who translated the questionnaire from English into Indonesian and back?
11. How long did it take for the respondents to complete the questionnaire?
12. Can the author(s) indicate the period in which the data were collected?
13. Can the author(s) clarify how the verbal consent was carried out?
14. How many questionnaires were distributed in person, and how many were carried out online?
15. The arguments raised by the author(s) on PLS in Section 3, should be cited. E.g., Partial Least Squares (PLS) via SmartPLS version 4.0 M3. PLS, a variance-based Structural Equation Modeling (SEM) method, is suitable for addressing issues like small sample sizes, missing data, and multicollinearity. The advantages of PLS include its applicability in theory development, capability to model multiple dependent and independent variables, and robustness against non-normal data.
16. The author(s) should avoid repetition in their paper. E.g., "A pilot test involving at least 30 questionnaires was conducted to assess the validity and reliability of the instrument, targeting postgraduate students from the Faculty of Economics and Business at Universitas Brawijaya. Data analysis for hypothesis testing utilized Partial Least Squares (PLS) via SmartPLS version 4.0 M3." " Before distributing the questionnaire, a pilot test was conducted to ensure the clarity and adequacy of the items. This pilot test involved 30 postgraduate students from the Faculty of Economics and Business at Brawijaya University."
17. The author(s) should provide a table indicating the measurement model and the discriminant validity
18. The author(s) should try their best to interpret the results and link them to their research objectives and existing literature.
19. The author(s) should clearly present the study's implications.

Is the work clearly and accurately presented and does it cite the current literature?

Partly

If applicable, is the statistical analysis and its interpretation appropriate?

No

Are all the source data underlying the results available to ensure full reproducibility?

Partly

Is the study design appropriate and is the work technically sound?

No

Are the conclusions drawn adequately supported by the results?

Partly

Are sufficient details of methods and analysis provided to allow replication by others?

No

Reviewer Expertise:

Trust, AI, Organizational Climate, Innovation Management

I confirm that I have read this submission and believe that I have an appropriate level of expertise to confirm that it is of an acceptable scientific standard, however I have significant reservations, as outlined above.
